# Efficacy of androgen receptor signaling inhibitors in combination with androgen deprivation therapy for castration-sensitive metastatic prostate cancer: a retrospective analysis in a Japanese cohort

**DOI:** 10.1007/s10147-024-02670-5

**Published:** 2024-12-18

**Authors:** Minekatsu Taga, Takeshi Sasaki, Shinichiro Higashi, Shoichi Kimura, Atsuro Sawada, Katsuki Tsuchiyama, Takahiro Inoue, Toshiyuki Kamoto, Naoki Terada

**Affiliations:** 1 Department of Urology, Faculty of Medical Science, University of Fukui, 23-3 Matsuoka-shimoaizuki, Eiheiji-cho, Yoshida-gun, Fukui, 910-1193 Japan; 2 Department of Nephro-Urologic Surgery and Andrology, Mie University Graduate School of Medicine, Tsu, Japan; 3 Department of Urology, University of Miyazaki, Miyazaki, Japan

**Keywords:** Metastatic prostate cancer, Androgen deprivation therapy, Androgen receptor signaling inhibitor, Overall survival, Castration-resistant prostate cancer

## Abstract

**Background:**

This study aimed to evaluate the efficacy of androgen receptor signaling inhibitors (ARSIs) combined with androgen deprivation therapy (ADT) for treating castration-sensitive metastatic prostate cancer in Japanese patients, focusing on the effects on time to the development of castration-resistant prostate cancer (CRPC) and overall survival (OS).

**Methods:**

This retrospective muti-institutional analysis included 332 patients diagnosed with metastatic prostate cancer in Japan between 2018 and 2023. The patients were categorized into two groups: patients receiving ADT combined with ARSI (ARSI group) and those receiving ADT alone or with bicalutamide (ADT group). Data on demographics, treatments, and outcomes were compared using the Kaplan–Meier method with propensity score matching.

**Results:**

We found an increasing trend in ARSI use over time. The median time to CRPC was significantly longer in the ARSI group than in the ADT group (47.1 vs. 15.2 months, *p* < 0.001); however, no significant differences in OS were observed before or after propensity score matching. The 1-year-survival rate of patients in the ARSI group tended to be higher than that of patients in the ADT group in subgroups with high tumor volume (96.1% vs. 85.0%) and high Gleason grade (98.1% vs. 85.9%).

**Conclusions:**

Adding ARSI to ADT extended the time to CRPC but did not significantly affect OS. However, it potentially suppressed the short-term risk of death in high-risk subgroups. This study highlights the need for further research to explore the characteristics of Japanese patients with metastatic prostate cancer in whom upfront ARSIs are effective.

**Supplementary Information:**

The online version contains supplementary material available at 10.1007/s10147-024-02670-5.

## Introduction

Metastatic prostate cancer is among the leading causes of cancer-related deaths in men, making early detection and treatment both a social and medical priority [1]. Traditionally, androgen deprivation therapy (ADT) with medical or surgical castration has been the cornerstone treatment for metastatic prostate cancer. However, most patients acquire resistance to castration therapy resulting in castration-resistant prostate cancer (CRPC). Androgen receptor signaling inhibitors (ARSIs) were introduced as a treatment option for CRPC to improve patient prognosis [2, 3]. In recent years, adding ARSIs to ADT as an initial treatment has been shown to improve the survival of patients, and the use of ARSIs as first-line treatment for metastatic prostate cancer has become common [4–6].

However, Japanese patients with metastatic prostate cancer are generally considered to have a good prognosis [7]. Therefore, the efficacy of adding ARSIs to ADT in Japanese patients with metastatic prostate cancer remains unclear. This uncertainty poses a significant barrier to optimizing treatment choices and strategies for individual patients. Therefore, in this study, we aimed to retrospectively analyze the effects of combining ARSIs with ADT on the prognosis of patients with metastatic prostate cancer across institutions in Fukui, Mie, and Miyazaki. Our objective was to provide insights into the efficacy of ARSIs in Japanese patients and to contribute to the advancement of personalized medicine for treating metastatic prostate cancer.

## Materials and methods

### Study population

In this study, we used data from patients diagnosed with metastatic prostate cancer between 2018 and 2023 at the University of Miyazaki, Mie, and Fukui Hospitals and other associated hospitals. This study was approved by the Institutional Review Board of each institute. The approval number for the University of Fukui Hospital was 20,230,132. Informed consent was obtained through an opt-out process. All patients had pathologically proven adenocarcinoma of the prostate, and extra-regional lymph node or distant metastasis was detected using computed tomography or bone scan at the time of diagnosis. Patient backgrounds and survival data were retrospectively obtained from medical records. The data included patient demographic and clinical information (age; prostate-specific antigen [PSA] level at diagnosis; Gleason grade group; Tumor, Node, Metastasis [TNM] classification; and metastatic sites) and treatment details. Metastatic burden was defined as high or low volume based on the Chemohormonal Therapy Versus Androgen Ablation Randomized Trial for Extensive Disease in Prostate Cancer (CHAARTED) classification system (high volume: presence of visceral metastases or four or more bone lesions with at least one beyond the vertebral bodies and pelvis; low volume: all others) [8]. All patients underwent ADT with a luteinizing hormone-releasing hormone (LHRH) agonist, an LHRH antagonist, or surgical castration. The ARSIs included abiraterone plus prednisolone, enzalutamide, or apalutamide. The ARSI used was at the discretion of the attending physician. Patients who received treatment with ARSI at the same time as or within 3 months of ADT initiation were categorized into the ARSI group. Patients who received ADT monotherapy or ADT plus bicalutamide as first-line therapy and who did not receive ARSI within 3 months of ADT initiation or before the development of treatment resistance to ADT were categorized into the ADT group. Resistance to ADT, with or without ARSI administration, was defined as CRPC. The duration from ADT initiation to CRPC onset (time to CRPC) and overall survival (OS) were examined. Treatment resistance was defined as PSA, radiological, or clinical progression. PSA progression was defined as an increase of 25% in PSA levels from the nadir, resulting in levels > 2.0 ng/ml [9]. When the therapy was changed before treatment resistance, the time to change the therapy was defined as CRPC onset.

### Statistical analysis

Data are presented as medians and ranges. Time to CRPC and OS were compared using a two-sided log-rank test with the Kaplan–Meier method. Baseline characteristics were compared between the groups using Fisher’s exact test and the Mann–Whitney U test. To match the patients’ backgrounds between the groups, propensity score matching was performed using a caliper for neighborhood-based estimation; the propensity scores used were calculated using logistic regression models with the following parameters: age, PSA level, clinical TNM stage, Gleason grade group, and metastatic volume. Thereafter, time to CRPC and OS were compared in all patients and between subgroups based on Gleason grade group (5 vs. ≤ 4) and metastatic volume (high vs. low). The 1- and 2-year survival rates were also compared. All statistical analyses were performed using EZR (Saitama Medical Center, Jichi Medical University, Saitama, Japan), a graphical user interface for R (R Foundation for Statistical Computing, Vienna, Austria). Values of *p* < 0.05 was considered statistically significant.

## Results

**Fig. 1:**
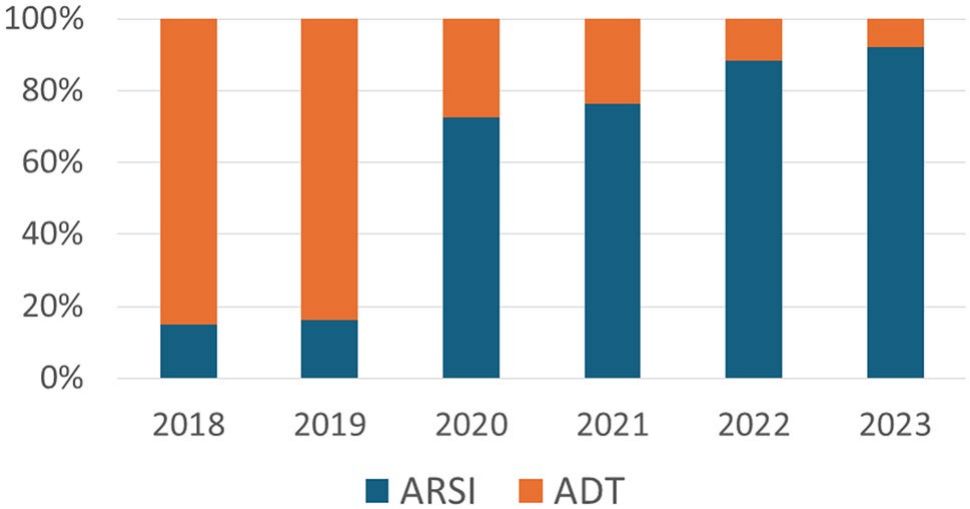
Number of patients receiving ADT plus ARSI (ARSI group) and those receiving ADT alone or ADT plus bicalutamide (ADT group) in each period from treatment initiation for metastatic prostate cancer. *ADT* androgen deprivation therapy, *ARSI* androgen receptor signal inhibitor

A total of 332 patients with metastatic prostate cancer were included in this study. Among them, 184 and 148 patients were assigned to the ARSI and ADT groups, respectively. The number of patients in the ARSI group increased annually (Fig. 1). The median age of the patients was 72 years (range 44–89) in the ARSI group and 75 years (range 52–90) in the ADT group; patients were significantly younger in the ARSI group than in the ADT group (*p* < 0.001). The median PSA level at diagnosis was 299 ng/ml (range 0.95–15,030) in the ARSI group and 249 ng/ml (range: 4.47–122,875) in the ADT group, with no significant difference between the groups (*p* = 0.303). The rate of visceral metastases was 27.1% (50 of 184 cases) in the ARSI group and 7.4% (11 of 148 cases) in the ADT group; this rate was significantly higher in the ARSI group than in the ADT group (*p* < 0.001). No significant differences in the clinical T and N stages or Gleason grade groups were observed between the groups (Table 1).

**Table 1:** Patient characteristics in ARSI and ADT group

	ARSI	ADT	*p*
Number	184	148	
Median (range) age (years)	72 (44–89)	75 (52–90)	< 0.001
Median (range) initial PSA (ng/ml)	299 (0.95–15,030)	249 (4.47–122,875)	0.303
T stage			
2	25	22	0.28
3	93	80	
4	62	36	
NA	0	10	
N stage			
0	65	50	1
1	118	93	
NA	0	5	
M stage			
1a	8	16	< 0.001
1b	126	121	
1c	50	11	
Gleason grade group			
2	2	2	0.85
3	12	12	
4	53	38	
5	109	78	
NA	8	18	
Metastatic volume			
High	136	82	< 0.001
Low	48	66	

*ADT* androgen deprivation therapy, *ARSI* androgen receptor signal inhibitor, *PSA* prostate-specific antigen, *T* tumor, *N* node, *M* metastasis

**Fig. 2:**
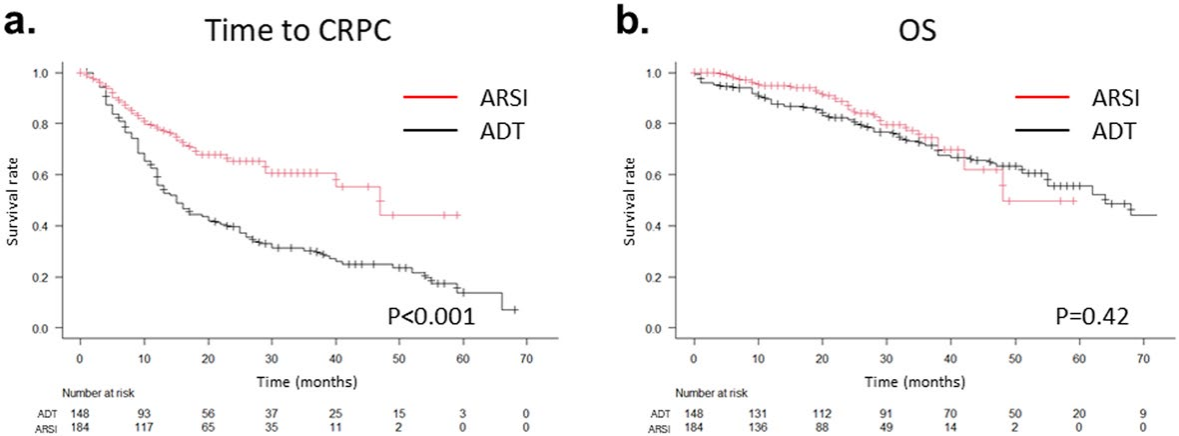
Kaplan–Meier curves of the time to CRPC (**a**) and OS (**b**) in all patients in the ARSI and ADT groups. The *p* values were determined using log-rank tests. *CRPC* castration-resistant prostate cancer, *OS* overall survival, *ADT* androgen deprivation therapy, *ARSI* androgen receptor signal inhibitor

In the ARSI group, 100 (54%) patients received abiraterone plus prednisolone, 57 (31%) received enzalutamide, and 27 (15%) received apalutamide. In the ADT group, 136 patients (91.9%) received bicalutamide in addition to ADT. The median time to CRPC was 47 months in the ARSI group and 15 months in the ADT group; the duration was significantly longer in the ARSI group than in the ADT group (*p* < 0.001). The median OS was 48 months in the ARSI group and 64 months in the ADT group, with no significant difference between the groups (*p* = 0.42) (Fig. 2).

**Fig. 3:**
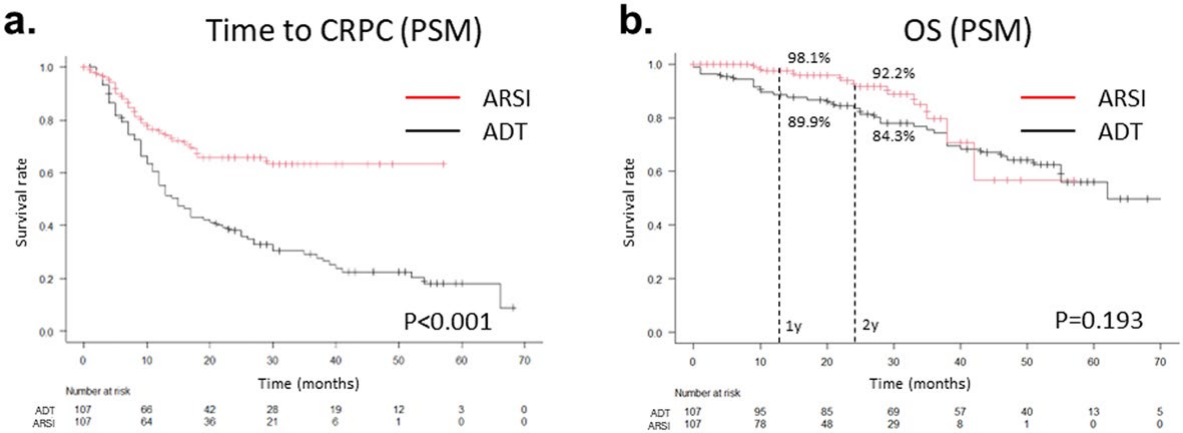
Kaplan–Meier curves of the time to CRPC (**a**) and OS (**b**) after propensity score matching in the ARSI and ADT groups. The *p* values were determined using log-rank tests. *CRPC* castration-resistant prostate cancer, *OS* overall survival, *ADT* androgen deprivation therapy, *ARSI* androgen receptor signal inhibitor, *PSM* propensity score matching

Propensity score matching was performed to reduce selection bias between the ARSI and ADT groups. Consequently, the backgrounds of the patients were not significantly different between the ARSI (*n* = 107) and ADT (*n* = 107) groups (Table 2). The median time to CRPC was not reached in the ARSI group but was 15 months in the ADT group; the time was significantly longer in the ARSI group than in the ADT group (*p* < 0.001). The median OS was not reached in the ARSI group but was 62 months in the ADT group, and no significant difference was observed in the log-rank test (*p* = 0.193). Conversely, the 1- and 2-year survival rates were higher in the ARSI group than in the ADT group (98.1% vs. 89.9% and 92.2% vs. 84.3%, respectively) (Fig. 3).

**Table 2:** Patient characteristics in ARSI and ADT group after propensity score matching

	ARSI	ADT	*p*
Number	107	107	
Median (range) age (years)	75 (44–89)	74 (52–90)	0.934
Median (range) initial PSA (ng/ml)	299 (0.95–15,030)	249 (4.47–122,875)	0.756
T stage			
2	14	20	0.492
3	64	57	
4	29	30	
N stage			
0	44	42	0.856
1	63	65	
M stage			
1a	7	8	0.856
1b	88	90	
1c	12	9	
Gleason grade group			
2	2	2	0.708
3	8	4	
4	28	29	
5	69	72	
Metastatic volume			
High	43	39	0.673
Low	64	68	

*ADT* androgen deprivation therapy, *ARSI* androgen receptor signal inhibitor, *PSA* prostate-specific antigen, *T* tumor, *N* node, *M* metastasis

**Fig. 4:**
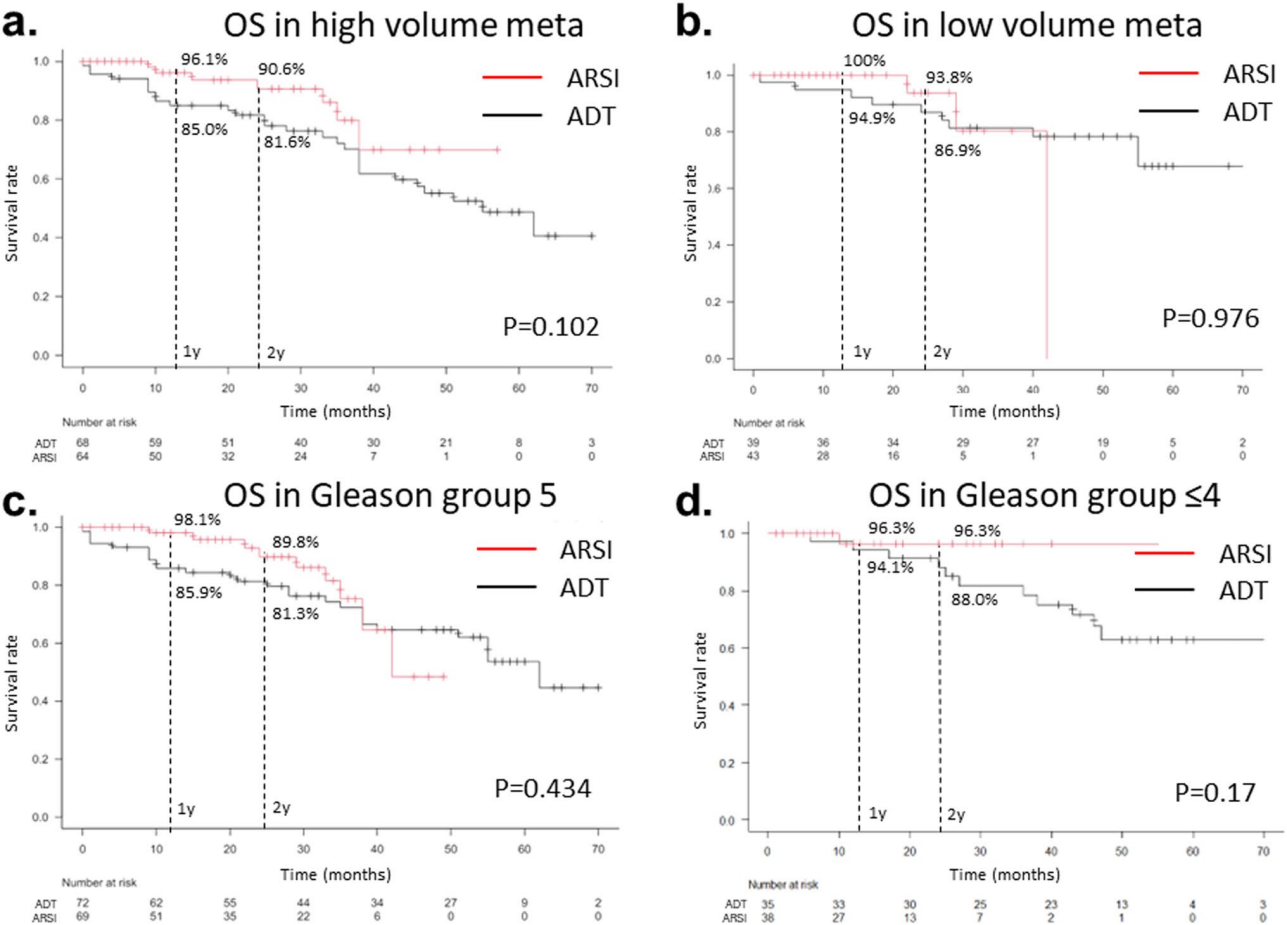
Kaplan–Meier curves of OS after propensity score matching in patients with low metastatic volume (**a**), high metastatic volume (**b**), Gleason grade group 5 (**c**), and Gleason grade group ≤ 4 (**d**) in the ARSI and ADT groups. The *p* values were determined using log-rank tests. The 1- and 2-year cumulative survival rates were determined in each group. *OS* overall survival, *ADT* androgen deprivation therapy, *ARSI* androgen receptor signal inhibitor

Patients were subcategorized based on risk categories in order to identify those in whom upfront ARSI added to ADT improved survival. They were classified into groups with high- and low-volume metastasis according to the CHAARTED criteria; the OS was not significantly different between patients in the ARSI and ADT groups in the log-rank test in these subgroups (*p* = 0.102 and *p* = 0.976, respectively). The 1- and 2-year survival rates tended to be higher in patients in the ARSI group than in those in the ADT group in both the high-volume (96.1% vs. 85.0% and 90.6% vs. 81.6%, respectively) and low-volume metastasis subgroups (100% vs. 94.9% and 93.8% vs. 86.9%, respectively). The difference in the 1-year survival rate was higher in the high-volume subgroup (11.1%) than in the low-volume subgroup (5.1%). Patients were also classified into subgroups based on Gleason grade groups of 5 and ≤ 4. In both these subgroups, OS was not significantly different between patients in the ARSI and ADT groups in the log-rank test (*p* = 0.434 and *p* = 0.17, respectively). The 1- and 2-year survival rates tended to be higher in patients in the ARSI group than in those in the ADT group in both the Gleason grade group 5 (98.1% vs. 85.9% and 89.8% vs. 81.3%, respectively) and Gleason grade group ≤ 4 (96.3% vs. 94.1% and 96.3% vs. 88.0%, respectively) subgroups. The difference in the 1-year survival rate was higher in the Gleason grade group 5 (12.2%) than in the Gleason grade group ≤ 4 (2.2%) (Fig. 4).

## Discussion

In this study, we compared treatment effects between patients who received ADT plus ARSI and those who received ADT alone or ADT plus bicalutamide by adjusting for background factors using propensity score matching. The time to CRPC was significantly longer with ADT plus ARSI than with ADT alone or ADT plus bicalutamide; however, no significant differences were observed in OS. In addition, no significant difference was observed when analyzing the high- and low-volume subgroups and high- and low-Gleason grades subgroups separately. These findings suggest that adding ARSI to ADT prolongs the time to CRPC; however, it does not consistently affect OS. Interestingly, these results are not consistent with those of a globally conducted randomized control trial (RCT) that demonstrated the efficacy of ARSI added to ADT in treating patients with castration-sensitive metastatic prostate cancer [4–6].

The results of retrospective studies conducted to determine the efficacy of ARSIs in Japanese patients with castration-sensitive metastatic prostate cancer were reported after 2021. Patients with LATITUDE high-risk disease (meeting at least two of the following three criteria: (i) Gleason score ≥ 8, (ii) presence of ≥ 3 lesions on bone scan, and (iii) presence of measurable visceral lesions) receiving ADT plus abiraterone were compared with those previously treated with ADT plus bicalutamide. Two studies did not report a significant difference in OS [10, 11]. A study that focused only on CHAARTED high-volume disease reported a significantly prolonged OS in patients treated with ADT plus abiraterone or docetaxel [12]. Another study indicated that treatment with ADT plus abiraterone resulted in prolonged OS only in patients with Gleason grade 5 primary lesions [13]. In the largest retrospective multi-institutional study in Japan, named the J-ROCK study, OS was significantly longer in patients receiving any ARSI added to ADT than in those receiving bicalutamide added to ADT; however, the patient backgrounds were not matched [14]. Another study suggested that patients treated with ADT and apalutamide may have better OS than patients treated with bicalutamide alone [15].

These retrospective studies indicate that the efficacy of ARSI in combination with ADT in Japanese patients with castration-sensitive metastatic prostate cancer is controversial. In our retrospective multi-institutional study, OS was not significantly different between the ARSI and ADT groups. An important factor that could explain the lack of observed differences is the approach to treatment initiation. In the ADT group, PSA levels were regularly monitored and ARSI treatment was initiated at the first indication of any increase in PSA. In the global RCT, ARSI treatment was delayed until radiological progression occurred in the ADT group to evaluate radiological progression-free survival. Approximately 90% of patients started ARSI treatment only in PSA progression without radiological progression in ADT group, although the accurate number of patients was not examined in this study. This practice may have mitigated the potential benefits of initiating ADT plus ARSI from the outset, thereby influencing study outcomes.

Global RCTs conducted to determine the efficacy of abiraterone (LATITUDE), apalutamide (TITAN), and enzalutamide (ARCHES) included Japanese patients and reported on the results of subgroup analyses in these patients. All the studies clearly revealed the efficacy of ARSIs in improving OS in all patient subgroups [4–6]. However, no significant improvement in OS was observed in the Japanese subgroup analyses [16–18]. One reason for this discrepancy could be the inclusion of small number of patients in the Japanese cohort. Another reason is that the OS in the ADT group in the Japanese cohort was better than that in the global cohort. Therefore, the addition of ARSI might not have prolonged the OS further. Previous reports have indicated that patients with metastatic prostate cancer in Japan have better prognoses than those of patients in the United States [7]. The efficacy of ADT for treating prostate cancer is higher in Asians than in Caucasians [19]. Despite advances in treatment strategies, the question regarding whether Japanese patients, who are known to have a better prognosis with ADT, benefit equally from the addition of ARSI to treatment regimens remains unanswered. This gap not only highlights the need for further investigations but also underscores the potential for gaining novel insights into personalized cancer treatment strategies.

As shown in Fig. 2, the median OS tended to be shorter in the ARSI group than in the ADT group (48 months and 64 months, *p* = 0.42), although the time to CRPC was significantly longer in the ARSI group than in the ADT group. It was suggested that the death from causes other than prostate cancer was higher in the ARSI group. Therefore, the cancer-specific survival was evaluated. Although the median cancer-specific survival time was not reached in both ARSI group and ADT group, the Kaplan–Meier curves show similar trends with those of OS (Supplemental Fig. 1). These results indicated that most of the death in the ARSI group was caused by prostate cancer. The reasons for the discrepancies between time to CRPC and OS might be the differences in the follow-up duration between the groups. The follow-up duration was not enough for evaluating the median OS in the ARSI group.

To explore the characteristics of Japanese patients with metastatic prostate cancer in whom the addition of ARSI was effective, OS was compared in the subgroups based on metastatic volume and Gleason grade between the ARSI and ADT groups after propensity score matching. No significant differences were observed between the high- and low-volume subgroups or between the high and low Gleason grade subgroups. However, the Kaplan–Meier survival curves indicated that the survival rate tended to be better at the short-term follow-up after treatment initiation. Therefore, we compared the 1- and 2-year survival rates. The 1-year survival rate was 6% higher in the high-volume group than in the low-volume group (11.1% vs. 5.1%) and 10% higher in the high Gleason grade group than in the low Gleason grade group (12.2 vs. 2.2%). These results indicate that the addition of ARSI may suppress the short-term risk of death in high-risk subgroups of Japanese patients with metastatic prostate cancer, although the difference was not significant in log-rank test.

This study has several limitations. Because of the retrospective multi-institutional nature of the study, treatment selection, including ADT or ADT plus ARSI and the type of ARSI (abiraterone, apalutamide, or enzalutamide), for each patient differed among institutes and was based on the physician’s preferences. Due to the observational nature of this study, biases originating from unmeasured covariates could not be completely eliminated. In comparing the differences between the treatment groups, especially in the subgroup analyses, the sample size was insufficient to draw definitive conclusions. Moreover, the follow-up duration is not enough to compare the OS in this study. To see the efficacy of ARSI to prolong the prognosis of metastatic prostate cancer patients, further study is needed with longer follow-up duration. However, this is the first study to show the efficacy of adding ARSI to the treatment regimen in subgroups of Japanese patients with metastatic prostate cancer based on metastatic volume and Gleason grade. The results may help in deciding between starting with ADT and adding ARSI at the time of PSA increase or starting with ADT plus ARSI as the first-line therapy for metastatic prostate cancer in Japan.

## Supplementary Information

Below is the link to the electronic supplementary material.

Supplementary file1 Supplement Fig. 1 Kaplan–Meier curves of cancer-specific survival (a) and that after propensity score matching (b) in the ARSI and ADT groups. The p values were determined using log-rank tests. Abbreviations: ADT, androgen deprivation therapy; ARSI, androgen receptor signal inhibitor; PSM, propensity score matching (TIF 91 KB)

## Data Availability

The datasets generated during and/or analyzed during the current study are available from the corresponding author on reasonable request.
