# Supplementary figures and images for: Efficacy of androgen receptor signaling inhibitors in combination with androgen deprivation therapy for castration-sensitive metastatic prostate cancer: a retrospective analysis in a Japanese cohort
Source: Int J Clin Oncol. 2024 Dec 18;30(2):351–7. doi: 10.1007/s10147-024-02670-5 (PMC11785660; doi:10.1007/s10147-024-02670-5)

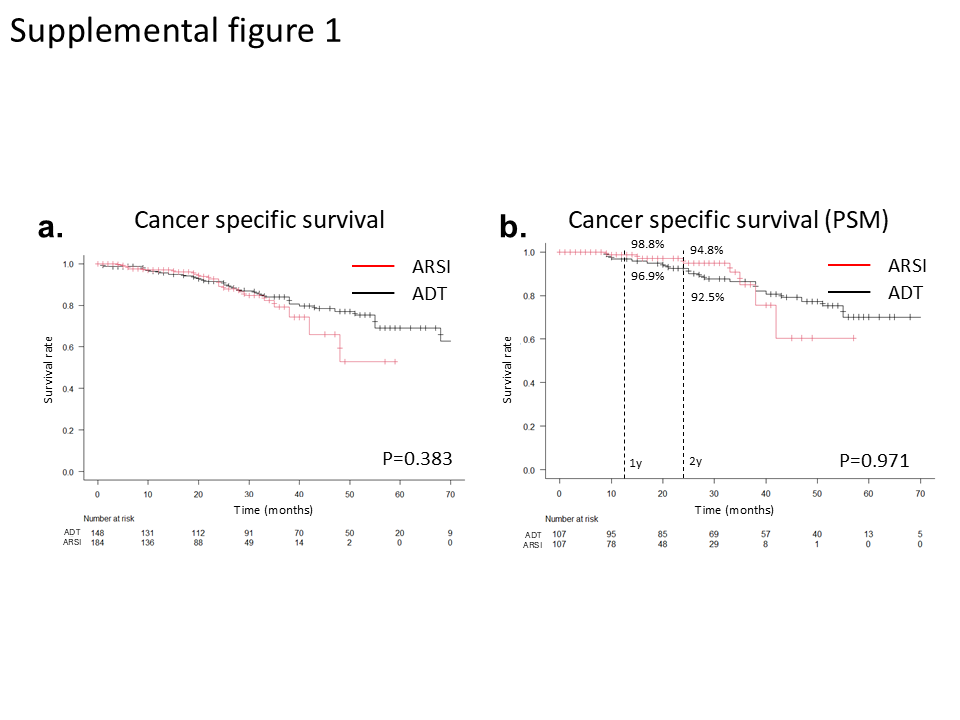

Supplement: Supplementary file 1 — Supplementary file1 Supplement Fig. 1 Kaplan–Meier curves of cancer-specific survival (a) and that after propensity score matching (b) in the ARSI and ADT groups. The p values were determined using log-rank tests. Abbreviations: ADT, androgen deprivation therapy; ARSI, androgen receptor signal inhibitor; PSM, propensity score matching (TIF 91 KB) [file 10147_2024_2670_MOESM1_ESM.tif]
